# The ethics of positive thinking in healthcare

**DOI:** 10.18502/jmehm.v12i18.2148

**Published:** 2019-12-21

**Authors:** Gabriel Andrade

**Affiliations:** *Assistant Professor, College of Medicine, Ajman University, United Arab Emirates.*

**Keywords:** Positive thinking, Self-help, Cancer, Healthcare, Positive psychology

## Abstract

In continuation with the New Thought movement that arose in the United States in the 19^th^ Century, there is now a massive self-help industry that markets books and seminars. This industry has also extended to healthcare in the form of positive thinking, i.e., the idea that happy thoughts are essential for health. While some of these claims may seem reasonable and commonsensical, they are not free of problems. This article posits that positive thinking has some ethical underpinnings. Extreme positive thinking may promote alternative forms of medicine that ultimately substitute effective treatment, and this is unethical. The emphasis on positive thinking for cancer patients may be too burdensome for them. Likewise, unrestricted positive thinking is not necessarily good for mental health. After considering the ethics of positive thinking, this article proposes a more realistic approach.

## Introduction

*Pessimism and Optimism as Philosophies*

**“**Positive thinking” suggests that in order to accomplish good things and pursue happiness, human beings must constantly have positive thoughts and prevent negative thoughts from entering their mind. This can be done by envisioning success, repeating constant affirmations, continuously saying good things to oneself in order to build self-esteem, and arousing happy thoughts that may block stressing ideas from one’s mind.

In the last three decades, this trend has been massively popular in psychology, and it has also been extended to healthcare and the business world. Positive thinking is also the foundation of the self-help industry, which is estimated to make around 10 billion US$ every year in products such as books and seminars. The healthcare industry has also borrowed ideas from this movement by assuring patients that their prognosis will be dramatically improved if they think positive thoughts.

Although it may seem like a recent phenomenon (with all the marketing techniques of advanced capitalism), in fact, positive thinking is a very ancient movement, long defended by many philosophers. Some philosophers are optimists; others are pessimists. The dialectic between optimism and pessimism is an ancient one, and to a certain extent, discussions about the merits of positive thinking in healthcare and elsewhere reflect this ancient dialectic.

Pessimist philosophers are prone to see the world as a hopeless place. Perhaps the most notorious of all pessimist philosophers, Arthur Schopenhauer, presented this view in no ambiguous terms: “Human life must be some kind of mistake. The truth of this will be sufficiently obvious if we only remember that man is a compound of needs and necessities hard to satisfy; and that even when they are satisfied, all he obtains is a state of painlessness, where nothing remains to him but abandonment to boredom. This is direct proof that existence has no real value in itself; for what is boredom but the feeling of the emptiness of life?” (1). Although he founded no school of philosophy, Schopenhauer had considerable influence over thinkers such as Nietzsche and Freud, both of whom also leaned towards grim understandings of human nature and the world. More recently, philosopher David Benatar is so disappointed with life, that he makes the ethical case for *not *having children: “Each one of us was harmed by being brought into existence. That harm is not negligible, because the quality of even the best lives is very bad – and considerably worse than most people recognize it to be. Although it is obviously too late to prevent our own existence, it is not too late to prevent the existence of future possible people” (2).

By contrast, religious thinkers tend to affirm the other end of the spectrum, i.e., optimism. In their view, inasmuch as the world is God’s creation, by necessity it is a good place. Obviously, there appear to be many imperfections in the world, but these philosophers typically go to great lengths in order to prove that those imperfections are actually part of a cosmic plan for a greater good. This approach, known as “theodicy”, attempts to justify God’s ways to man, and by doing so, attempts to present the world in an optimistic light. Perhaps the most notorious philosopher in this regard was Leibniz, who affirmed that this is by necessity “the best of all possible worlds” (3).

Leibniz’ brand of optimism was more cosmic, and it did not provide much solace to people. In his philosophy, the world may seem like a terrible place, but in fact, it is the best one there can ever be. By contrast, some American philosophers and religious authors were presenting a more radical form of optimism by the 19^th^ century. This school of thought came to be known as New Thought (4), and its main principle was that “our mental states are carried forward into manifestation and become our experience in daily living” (5). This basically means that our minds are sufficiently powerful to bring about whatever we desire, also presented in the phrase “mind over matter”. In religious terms, this was upheld by the Christian Science, a movement founded by Mary Baker Eddy, whose main thesis is that disease is fundamentally a state of mind that can be mentally reversed. 

In the late 20^th^ century, this approach was additionally upheld by New Age spirituality. New Age actually encompasses a wide variety of beliefs, but one of its recurring themes is the appeal to Hindu idealist traditions. According to these doctrines, the material world is an illusion, and it can be changed with the power of the mind. As we shall see, *The Secret*, a major promoter of positive thinking, relies on a variant of Hindu idealism adapted to Western cultural markets.

This is in fact a major shortcoming of positive thinking in the West. In non-Western cultures, there are numerous traditions that posit specific ideas about wellness. In the last century, these ideas have been introduced in Western cultures, and they have been applied in very different contexts from where they originally arose. The result has often been a misapplication of these ideas to entirely different cultural settings (such as Ayurvedic medicine in the West), with negligible results.

Schopenhauer’s gloomy approach is certainly not supportive of health. Although Schopenhauer was not apologetic about suicide, his pessimistic views about the human condition and the purpose of life may easily lead to neglect of health, for one may wonder what the point of healthy living is, if life is horrible anyways. Yet, the kind of optimism embraced by Leibniz and the New Thought movement is not without its problems. In fact, although Voltaire’s *Candide *is not an entirely accurate representation of Leibniz’s philosophy, he did raise a legitimate criticism: sometimes optimism can be as cruel as pessimism. To tell someone that they must cheer up in the face of tragedy because this is the best of possible worlds, or because in every crisis there is an opportunity, is despotic on its own.

Voltaire was not a pessimist. In fact, he seemed to lead a happy life, and he was confident that Enlightenment was guiding humanity through a good path (6). However, he was aware that optimism can be problematic as well, especially by compelling people to put a smile on their faces when the circumstances do not allow for it.

This middle-ground position ought to be defended as the philosophical approach in healthcare. Patients should be encouraged to cheer up as far as possible, because optimism can be a good strategy in healthcare. Physicians such as Patch Adams should be ethically praised for their efforts to bring joy to patients through comedy. But ethics also require that limits be placed on the positive thinking trend that is becoming increasingly popular in healthcare, since unrestricted optimism feeds magical thinking (as in mind-over-matter thinking), and the fostering of delusions can never be ethical. As it is usually the case with magical thinking, positive thinking encourages an excessive illusion of control (the tendency to overestimate the ability to control events), and this illusion can lead to unethical outcomes (7).

Furthermore, unrestricted positive thinking has significant downsides in terms of psychological development, cultivation of healthy habits and care of terminally ill patients, as well as detrimental, larger societal effects. In what follows, I shall explore the ethics of positive thinking in healthcare, pointing out what is reasonable and acceptable, and also highlighting how positive thinking in healthcare sometimes becomes unethical. I will work under the hypothesis that, with some exceptions, positive thinking has increasingly become unethical in healthcare, and healthcare professionals need to be aware of this in order to make the necessary corrections.

***Positive Thinking and General Health Effects***

For many centuries, people have had the intuition that stress has detrimental effects on health. Hans Selye’s studies began to empirically test this hypothesis, and his case was fairly convincing, even though there are some concerns about the motivations of his research (he was financed by tobacco companies, who were interested in placing guilt on stress for many diseases, so as to release tobacco of responsibility for health failings) (8). This was even more impressively confirmed by Robert Ader in a famous experiment (9). He fed rats with a combination of saccharin-laced water, and the drug Cytoxan to induce nausea and taste aversion. This combination induced classical conditioning in rats, who came to associate the saccharine-laced water with the drug. Afterwards, the rats were fed only the saccharine-laced water, but they died in great numbers nevertheless. Ader concluded that even without taking the drug, the rats’ immunological system had been suppressed with the mere stress of drinking water that had previously been associated with nausea through classical conditioning.

This experiment laid the basis for what has been called the field of psychoneuroimmunology. This discipline purports to study the interactions between the nervous system and the immune system, and as Ader’s experiment adequately proves, high levels of stress result in a diminished immunological response, and therefore greater exposure to sickness.

It is also true that placebo effects can be powerful, and may be effective in up to 30% of their applications (10). In that regard, encouraging patients to have constant positive thoughts may actually be helpful in the treatment of many conditions. However, the rate of effectiveness of placebos should actually be reduced by considering the Hawthorne effect, Rogers’s phenomenon, and the Simpson paradox (11). Furthermore, it must be kept in mind that not all diseases have a psychosomatic aspect, and in some diseases (asthma being the most notorious), placebos can actually be detrimental, for they may take care of the symptoms, but not the disease itself. This can be very dangerous, as the patient does not seek medical care because he/she feels fine, when in fact he/she is not (12). Moreover, the application of placebos also has ethical shortcomings, as it is a form of deceit, and this goes against the basic principle of informed consent (13). 

On the basis of the findings of psychoneuroimmunology, new theories have emerged. It is reasoned that if intense stress results in sickness, then most (if not all) immunological failings are due to lack of positive thinking. And, of all diseases interpreted through this lens, cancer occupies a central place. Thus, enthusiasts of positive thinking typically claim that cancer may appear as a result of too much stress, and positive thinking is a very efficient way of preventing it and even curing it. For example, in a book called *9 Steps to Reversing or Preventing Cancer and Other Diseases*, Shivani Goodman argues that this disease comes as a result of “toxic attitudes” and “emotional pain” (14). Physician Deepak Chopra argues that cancer can be cured by visualizing being well (15). Even an oncologist, Carl Simonton, argues that cancer happens as a result of a weakened immunological system (16), and inasmuch as the immunological system has a strong connection with the nervous system (as research in psychoneuroimmunology suggests), thoughts do play a significant role in this disease. 

There are many reasons to doubt all of these claims. It is beyond question that stress affects health negatively, but that does not imply that constant positive attitudes, unrestrained optimism, happy thoughts and reaffirmations are causes of good health. There certainly is a correlation between optimism and health. For example, optimism is related to a lower mortality rate (17), better standards of health and faster recovery rates in some diseases (18) and improved immunological response (19). We should not assume, however, that happiness and optimism cause good health, since the reverse may actually be the case: someone with a good immunological system seldom gets sick, and as a result, has better opportunities to be happy. In fact, some studies point in that direction (20). We simply do not know sufficiently well the direction in causality, and should not rush to conclusions.

Be that as it may, when it comes to cancer, there are greater doubts that a positive attitude can cure it, or that even the immunological system is involved. If the latter theory were true, common sense would indicate that patients with HIV are at greater risk of developing cancer, since the HIV virus attacks the immunological system; likewise, chemotherapy would strengthen patients’ immunological response. Both of these hypotheses are incorrect, and this points in the direction that the immunological system plays little role in cancer.

Thoughts and mental attitudes are even less relevant in the pathogenesis of cancer. Much has been made of alleged personality types being related to cancer (21), but this in fact has been debunked by more competent studies (22). Positive attitudes may be a factor in helping to cope with the burdens of cancer treatments, but they are not a factor in the treatment of cancer itself. These empirical data suggest that although the desirable effects of positive thinking on health may have some basis, this is not fully confirmed by science. In a scientific approach, promoters of positive thinking should follow a more cautious outlook, as the evidence is not entirely supportive of their claims.

***Cancer and Positive Thinking***

In principle, it makes sense to propose that positive thinking is a much-needed resource in the treatment of cancer. Patients struggle throughout the course of this disease, and in order to keep going, they need to find some meaning in their experience. In fact, finding meaning in the face of adversity is an important predictor of survivability. Victor Frankl’s famous memoir of his days in an extermination camp, *Man’s Search for Meaning*, makes a strong case that those who find meaning in things (even if they otherwise seem pointless), have greater probability of survival (23). That is why positive thinking has become such a major aspect of cancer treatment.

However, the way positive thinking is conducted with cancer patients has many ethical shortcomings. Very often, patients’ autonomy is violated. Sadness and stress in the face of adversity is a normal response, yet cancer patients are usually overly pressured *not *to feel sad. Joy becomes an obligation, and the concept of “mandatory fun” has an uncanny totalitarian aspect: patients are deprived of their emotional autonomy, and are forced to feel in a particular way.

The result is typically that, apart from the burden of dealing with the stress of cancer and the side effects of its treatment, patients now have the additional stress of having to be positive all the time. If they fail to do so (and naturally, most of them do, given the state of their condition), they feel additional sadness, for failing to meet the expectations. Cancer patients are not allowed to have autonomy in their feelings, and they end up being conducted by others about how they should feel. 

By having this autonomy removed, cancer patients are treated as underage subjects (precisely those that, in standard conditions, do not have autonomy). Barbara Ehrenreich went through this process as a cancer patient, and in her investigation of the positive thinking culture, she provides many examples of the infantilization of cancer patients (24). For example, breast cancer patients are offered pink ribbons and teddy bears, as objects to encourage positive attitudes. Other objects frequently distributed to breast cancer patients are boxes of crayons, pink objects, perfumes, body creams, and many other items typically reserved for children. Ehrenreich also makes the observation that this kind of infantilization is typical in breast cancer patients, but not in, say, prostate cancer patients. She therefore sees some patriarchal overtones in the positive thinking movement, arguing that men see female cancer patients as less than adults.

Positive thinking is frequently forced on cancer patients in the form of group therapy. Patients are encouraged to support each other by sharing their experiences and offering encouragement. Of course, there is nothing intrinsically wrong with this approach. However, the way these group therapies are framed makes it too invasive for the patient. The peer pressure coming from the group, overly encouraging the patient to keep a positive mind frame, ends up placing excessive stress on the patient, who feels that his/her sense of autonomy is eroded. In fact, there are studies that show that support groups do not increase survival rates amongst cancer patients (25). Even David Spiegel, who had originally published prominent research advocating support group therapy for the treatment of cancer, later on agreed that those therapeutically efforts offer no significant results, and concluded that cancer survival is not influenced by a patient’s emotional status (26). In fact, there is hard data suggesting that women who see cancer as something positive (as in the title of one such book, *The Gift of Cancer*), end up having worse mental functioning (27). 

Group therapy for cancer patients has other ethical shortcomings. The positive thinking movement has made strides in the business world and in self-help literature. One particular persistent idea in those settings is the ostracization of negative people, as part of a strategy for positive thoughts. Self-help books frequently contain passages such as this one: “That may sound harsh, but the fact is that negative people do suck. They suck the energy out of positive people like you and me. They suck the energy and life out of a good company, a good team, a good relationship…. Avoid them at all costs. If you have to cut ties with people you’ve known for a long time because they’re actually a negative drain on you, then so be it. Trust me, you’re better off without them” (28). On the basis of this idea, cancer support groups occasionally opt to expel those patients that have metastasis (29). The rationale is that these patients bring too much negativity to the group, and thus represent a danger to it. It is not hard to see the ethical problems with these procedures. Patients with metastasis are unfairly discriminated against, which goes against the ethical principle of justice. Furthermore, they are abandoned and withdrawn from the group, precisely at the moment when they may need the greatest support.

This excessive emphasis on positive thinking amongst cancer patients often leads to physicians giving false assurances to patients. Again, this is ethically problematic, as false assurances violate the principle of autonomy by depriving the patient of a full opportunity for informed consent (30). 

 More deeply unethical are those therapies that urge cancer patients to abandon conventional treatments and opt for alternative methods that rely entirely on positive thinking. The more radical varieties of positive thinking ultimately go back to the mind-over-matter approach of New Thought. One particularly recent trend in this movement is the so-called “law of attraction”, made popular by the best-selling book and film, *The Secret. *According to this law, thoughts attract realities. Therefore, if someone is diagnosed with cancer, all he/she has to do is to think hard about getting better, and those thoughts have the power to attract health and make cancer go away. With this reasoning, no conventional therapy is needed. 

Needless to say, the “law of attraction” is a form of very naïve magical thinking, which borders on being delusional. Parapsychologists have long been interested in studying how the mind may be able to move matter remotely (psychokinesis), but all studies have failed in coming up with evidence for these claims (31). As often happens with promoters of magical thinking in recent decades, these ideas are usually covered with a superficial veneer of scientific-sounding jargon. Thus, proponents of the “law of attraction” usually claim that quantum physics proves that the mind has the power to actualize thoughts via remote influences on matter. This is in fact a very misguided interpretation of quantum entanglement (32). 

TV broadcaster Oprah Winfrey made sure *The Secret *would get massive readership. Eventually, many viewers decided to stop conventional treatment for curable cancer altogether based on the tenets of *The Secret *(33). Most ethicists agree that alternative and folk medicine is mostly harmless, and therefore medical students are encouraged to respect patients’ cultural worldviews. However, alternative medicine becomes a problem when it substitutes evidence-based treatments. Many alternative medicine treatments rely on positive thinking, and in some cases, patients are encouraged to abandon conventional treatments, so as to fully dedicate to think positive thoughts. Ryke Geerd Hamer’s New Germanic Medicine was a movement that actively recommended patients to abandon science-based treatments in favor of mental cures (34). Hamer claimed that all diseases are controlled by the brain, and therefore their cure can be wholly mental. His approach to medicine was deeply unethical, and predictably, his medical license was removed, as many of his patients died from curable diseases. It is important, however, to understand that even if Hamer’s approach was extreme, it was still embedded in positive thinking, and his case is an illustration of the unethical variants that unrestrained positive thinking can lead to.

One further ethical problem with excessive emphasis on positive thinking is that it ultimately leads to victim-blaming. Inasmuch as cancer can be cured with positive thoughts, if someone is not cured or “loses the battle”, it must be because that person did not try hard enough to affirm himself/herself, or envision a cure. Thanks to the work of Melvin Lerner, we now know that the “just world” bias is deeply enshrined in the human mind (35). According to this way of thinking, people usually think that everyone gets what they deserve: virtuous actions are rewarded in life, and bad deeds are punished with misfortunes. Even though this is a very popular way of thinking, let us not forget that it is a bias. Bad things happen to good people, and vice versa. Regrettably, *The Secret *does not even have a moral dimension: according to this book’s thesis, disease comes, not necessarily to bad people, but to those who have not had positive thoughts. Ultimately, if they do not manage to get better, it is their own fault, for not wanting it and thinking about it hard enough. Extreme positive thinking eventually becomes a form of victim-blaming, and this is deeply unethical. As documented by Chaple et al., this kind of thinking leads to stigma and shame amongst patients, further adding to their unfortunate condition (36). 

***Positive Thinking and Mental Health***

In all the realms of healthcare, positive thinking has surely been the most enthusiastically upheld by mental healthcare providers. There is in fact a massive industry of books, films, seminars and even branding objects (caps, shirts, key chains, etc.) that repeat *ad nauseam *the tenets of New Thought and positive thinking, promising better results in terms of psychological well-being. This industry usually bypasses conventional approaches in psychotherapy (psychodynamic, cognitive behavioral, humanistic), and aims for the procurement of products that allow people to cope on their own, simply by reading books or listening to tapes. It is thus called the “self-help” industry.

Barbara Ehrenreich acutely observes that since psychopharmacology has made impressive advances in the last two decades, psychologists feel the threat of being left without much to do in the treatment of mental health. Thus, they now have an agenda in pushing positive thinking as the last resource of talking cures, thus strengthening the self-help empire.

We know that despite massive revenues, the self-help industry has been deeply ineffective, as the millennial generation is experiencing higher rates of anxiety and depression compared to the previous generations (37). In fact, investigative journalist Steve Salerno discovered that the most likely reader of a self-help book is a person who had previously bought a similar book not long before (38). The self-help industry executives seem to be fully aware that their products are ineffective, but they unethically remarket the same products over and over again, thus making huge profits. Indeed, self-help books are written in repetitive prose. Even during the heyday of one of the best-selling self-help books of all time (Norman Vincent Peale’s *The Power of Positive Thinking*), critics already noted that “…The chapters of his books could easily be transposed from the beginning to the middle, or from the end to the beginning, or from one book to another. The paragraphs could be shuffled and rearranged in any order” (39). These books are notoriously simplistic, and require little engagement from readers. They thus unethically contribute to the dumbing down of society. In fact, some self-help authors such as Jeffrey Gittomer explicitly recommend readers *not *to read or watch news, because they are too negative (40). Needless to say, this kind of willful ignorance of current events is detrimental to the adequate education of society.

Admittedly, some sectors in academia have presented a more respectable form of positive thinking and self-help. The so-called “positive psychology” school, founded by Martin Seligman, purports to focus on the development of the positive aspects of life, and aspires to document the benefits of happiness and optimism in health and life in general (41).

Nevertheless, even positive psychology is not without its problems. For one, its concepts are too elusive. Happiness is never truly satisfactorily defined by scholars of positive psychology. Although Seligman’s Authentic Happiness Inventory seems to have acceptable levels of validity and reliability (42), he purports to measure happiness in an equation which is ill-formulated (43).

 Be that as it may, the truth is that positive thinking has its shortcomings in mental health as well. Positive psychology has been additionally criticized for pushing the agenda to pathologize grief. The *Diagnostic Statistical Manual (DSM-5*) opted to remove the grief exclusion in the diagnosis of major depression, and this has met with strong criticism (44), for it places excessive pressure on patients to not feel sad, even when their loved ones have passed away.

Julie Norem has done extensive research documenting how some people may satisfactorily use pessimism as a defense mechanism. She proved how “individuals may sometimes use low expectations to cope with their anxiety so that it does not become debilitating… low expectations may help individuals negotiate risky situations by showing that interference with the defensive-pessimism strategy impairs performance” (45). Consequently, in people who use pessimism as an efficient defense mechanism, constant positive thinking may actually backfire, as subjects are left without a good resource to deal with anxiety.

In fact, Wood et al. have done important research showing that subjects who kept on making positive self-statements such as “I am a lovable person” or questioned the truth of that statement felt worse compared to those who did not repeat positive self-statements or did not concentrate on the fact that it was true and false at the same time (46). The key aspect is self-esteem. Positive thinking may work better in subjects with higher self-esteem, but in those with lower self-esteem, positive thinking is counterproductive, as subjects easily come to realize that they are engaging in self-deceit, and that leads to increased depression. In fact, hyper-optimism carries an additional risk of depression, for the hyper-optimist subject may not be adequately prepared in the event of a failure.

Excessive positive thinking may also lead to overconfidence in doing tasks, and consequently to poor performance. For evolutionary reasons, stress has an important physiological function, i.e., warning of dangers via the sympathetic nervous system and the fight-or-flight reaction. Admittedly, it is true that our current living conditions are different from the African savannah in which most of human evolution took place, but some measure of stress is needed for everyday living. Per the Yerkes-Dodson law, we know that there is an empirical relationship between arousal and performance that becomes manifest as a curve, and while too much anxiety is prejudicial for performance, too little is prejudicial as well (47). Positive affirmations may induce too much relaxation in a task, which may consequently be poorly done. In this regard, a stronger critique of positive thinking is warranted.

In fact, we know that negative thinking can make people more analytical so that they will engage more in critical thinking. These are skills that are required for good functioning and adequate mental health. In a review study Joseph Forgas reports that “negative affect can improve memory performance, reduce judgmental errors, improve motivation, and result in more effective interpersonal strategies” (48). One study by Shigehiro Oishi documents that moderately happy people are more successful professionally and economically than extremely happy people (49).

Likewise, Von Helversen et al. found that depressed subjects are better at decision-making (50). Daniel Kahneman has also done extensive studies showing that people may easily engage in optimism bias, and this may lead to a planning fallacy (thinking that tasks may require far less time), ultimately harming performance tasks (51). This may also have implications in physicians’ performances: one particular study showed that this kind of bias can lead to medical errors, thus affecting healthcare as a whole (52). Of course, this does not imply that depression is a good thing by itself, but it does suggest that positive thinking must have limits, because unrestrained optimism can be very distorting. 

Positive thinking has notorious prejudicial aspects in other areas related to healthcare. For example, overly positive people may neglect insurance coverage. People buy insurance thinking of adverse scenarios in advance. According to the “law of attraction”, this would be a sure way of inviting catastrophe, and therefore should be avoided. In countries with socialized medicine this is not necessarily a big problem, but in countries with no socialized medicine (such as the United States), this is a major hassle, for individuals that refused to think about adverse events may end up without adequate healthcare. 

One study shows that people who do not buy health insurance prefer to spend money on things like alcohol and tobacco (53). Surely, in the short term both tobacco and alcohol propitiate happier thoughts and positive thinking than risk assessments. This is related to the fact that positive thinking also tends to suspend delayed gratification. By insisting that one must pursue whatever makes one feel good in order to arouse positive thoughts, excessive positive thinking engages in a kind of destructive hedonism that completely disregards risks. It is thus no surprise that excessively optimistic people eat unhealthier foods (54) and have unprotected sex more often (55).

Academic positive psychology aptly warns that money cannot buy happiness and positive psychology scholars often state that lottery winners ultimately revert to their original happiness levels (56)); however, the more popular variants of positive thinking do emphasize the value of conspicuous consumption. The positive thinking movement is closely associated with the motivational drive that prevails in the sales and business world, and accumulation and display of wealth is an important aspect of this association. Self-help classic books such as Wallace Wattles’ *The Science of Getting Rich *or Napoleon Hill’s *Think and Grow Rich* insist on using positive affirmations as a way of getting rich. Failing to be rich (and to show it, of course) would be an indication of poor mental habits (i.e., not enough positive thinking). This also seems to be the message of the so-called “prosperity theology”, although with a religious twist: being rich is an indication of God’s favor, and therefore people must strive to be rich through positive thinking.

This excessive emphasis on conspicuous consumption and motivation to accumulate wealth can lead to very dysfunctional behaviors that ultimately result in impaired mental health. There is great self-entitlement in self-help literature and the positive thinking movement as a whole. For example, in the film version of *The Secret*, a woman desires a necklace exhibited in a store; she then concentrates on the thought of getting it and, lo and behold, the film shows her wearing it. We may be left wondering whether she robbed the store, just to accomplish her dreams. Motivational literature typically exhorts to go beyond limits; unfortunately, this may occasionally include going beyond moral limits.

 Excessive positive thinking also carries the risk of isolating subjects. For example, the self-help book *Secrets of the Millionaire Mind *recommends getting rid of negative people (57). It provides no opportunity whatsoever to improve relationships with negative people. Obviously, this kind of thinking induces divorce and straining of relationships with some people, just because they happen to not be as positive.

## Conclusion

Apart from the ethical shortcomings of positive thinking that directly affect patients, there are wider prejudicial effects in healthcare. Given its reliance on magical thinking (i.e., the “law of attraction”), the self-help industry incentivizes alternative medicine, and this has ethical problems of its own (58). Products that promote self-help and positive thinking are not effective, even though they constantly claim positive results. This kind of deceit is deeply unethical as well.

There are also concerns with positive thinking that go beyond healthcare. Excessive positive thinking may affect society in many ways, and this may ultimately lead to harmful epidemiological affects. For example, Ehrenreich makes a convincing case that the 2008 global financial collapse was deeply influenced by excessive optimism from brokers and bankers. Likewise, Makridakis and Moleskis aptly document how positive illusions facilitate wars, since politicians are too confident of victory and undermine risks (59), and that itself also leads to problems of public health.

None of this implies that one must assume a Schopenhauerian pessimism, and recommend it to patients in health practice. Happiness is important, and indeed, it can be sought. When it is achieved, happiness can certainly lead to better health. However, ancient philosophers long knew about the so-called “paradox of hedonism”: the more one tries to find pleasure, the less one finds it. Ethicists frequently warn that happiness cannot be sought directly (60). This is not entirely true, as for instance according to William James’ facial feedback hypothesis, smiling constantly does bring about greater happiness, and Botox injections may even be a treatment for depression (61). But it is important to note that this is about *doing *things to be happier, not just having positive thoughts and being irrationally optimistic, in the vain hope that thoughts will magically attract good things.

Be that as it may, the kind of happiness that is sought should also be based on a deeper philosophical insight. John Stuart Mill was certainly no pessimist, and his utilitarian philosophy is wholly about happiness and pleasure. But he was onto something when he argued that it is “better to be Socrates than a fool satisfied” (62); i.e., happiness is important, but what we should be after is a kind of higher and sublime happiness. Unfortunately, positive thinking induces more conspicuous consumption, as in self-help guru Marianne Williamson’s infamous “seek ye the Kingdom of Heaven, and the Maserati will get here” (63).

As Barbara Ehrenreich wisely points out, the alternative to positive thinking is not negative thinking, but rather realism. The world can be a nice place, but in those instances when it is not, it is pointless to delude ourselves and believe that just by thinking it is a good place, it will become so. This applies to patients and health practitioners, as adequate treatments come with adequate diagnoses, but positive thinking runs the risk of dispensing with them. To sum up, wishful thinking is a common logical fallacy, and it should be avoided.

 Likewise, ethics requires that patients be given the right to be sad. Urging people to be happy in the face of tragedy is a very oppressive act. Voltaire’s contempt for Leibniz’s optimism was precisely a protest against the insensitivity of people who believed they had found meaning and joy in things such as the 1755 Lisbon Earthquake. Voltaire demanded more empathy from people, so they could understand what sufferers go through. Medical ethics require the same from health practitioners.
